# Cottage Industries in Scotland

**Published:** 1889-10-19

**Authors:** 


### Cottage Industries in Scotland.

For the last four years the Marchioness of Stafford has been in the habit of holding a sale in Inverness of home-spun cloths, knitting, etc., made by the crofters in Sutherland. This year she arranged to hold the sale in London, and Lady Rosebery decided to join with her in exhibiting and selling the products from other parts of Scotland. The sale was held at Dudley House, on 3rd and 4th July, and was very successful. It is now proposed to form an association, entitled the Scottish Home Industries Association, to encourage and develop the industries of the Highlands, in order to give employment to a larger number of people and to stimulate a demand for home-made articles, which many people still maintain are superior to those made by machinery. Four central committees are being formed in Edinburgh, Glasgow, Inverness, and Aberdeen, and another for the management of Orkney and Shetland. These branch committees will receive subscriptions for the association, collect work, and hold local exhibitions and sales; and it is hoped to get the scheme widely known by next year. It is proposed to establish a central depot in London with a small staff, where patterns will be exhibited and orders taken, and the founders of the association think that by taking the cloths, etc., direct from the worker, they will sell more than has been done in years gone by, and at a cheaper rate, for they desire no profit themselves, and will only add 10 per cent. to the price of goods to defray expenses of carriage, etc. They propose also to send experts round the districts where industries exist, to improve the work, introduce new dyes for the cloth, and so to make an article which will find a market and give satisfaction. H.R.H. Princess Louise is patron of the association, and it has received much encouragement from the Prince and Princess of Wales.

